# Causal beliefs and behavioural self-evaluation in cardiac rehabilitation: a cross-national study in the United States and Japan

**DOI:** 10.1186/s12872-026-06452-x

**Published:** 2026-08-10

**Authors:** Tamami Shirai

**Affiliations:** Department of Radiology, School of Medicine, University of California San Diego, La Jolla, USA

**Keywords:** Causal attribution, Behavioural self-evaluation, Illness perception, Self-efficacy, Behaviour change, Cross-cultural comparison

## Abstract

**Background:**

Patients with established cardiovascular disease (CVD) continue to face substantial residual risk of recurrent events despite optimal medical management. Beliefs about disease causation, particularly attribution to genetic versus lifestyle factors, may influence health behaviour; however, their relationship with behavioural self-evaluation remains poorly understood. This study examined (1) cross-national differences in causal beliefs between patients in the United States (US) and Japan, and (2) associations between these beliefs, exercise confidence, and behavioural self-evaluation.

**Methods:**

A cross-sectional study was conducted among phase II cardiac rehabilitation patients in the US (*n* = 56) and Japan (*n* = 120). Causal beliefs, exercise confidence, and self-reported exercise behaviour were assessed by questionnaire. Between-group comparisons were performed using independent-samples *t*-tests and factorial ANOVA; associations between beliefs and behaviour were examined using binary logistic regression.

**Results:**

US participants attributed their CVD more strongly to genetic factors than Japanese participants (mean ± SD: 55.33 ± 32.06% vs. 29.36 ± 30.68%; Cohen’s *d* = 0.83, *p* < 0.001), whereas Japanese participants attributed their CVD more strongly to lifestyle factors (63.55 ± 29.62% vs. 41.82 ± 30.28%; Cohen’s *d* = 0.73, *p* < 0.001). US participants demonstrated greater confidence in achieving exercise goals than Japanese participants (*p* < 0.001). An exploratory interaction between disease type and genetic attribution was observed for exercise confidence (*p* = 0.03). Participants with high lifestyle attribution were less likely to report their current exercise level as sufficient (behavioural self-evaluation) (OR 0.46, 95% CI 0.22–0.97, *p* = 0.042).

**Conclusions:**

Patients’ beliefs about disease causation differ between the US and Japan and are associated with exercise confidence. The findings suggest that belief systems may represent an important psychological pathway underlying behavioural self-evaluation, with potential implications for cardiovascular secondary prevention. Importantly, beliefs may influence how patients evaluate the adequacy of their behaviour rather than behavioural engagement alone. Future longitudinal and intervention studies are warranted to determine whether interventions targeting causal beliefs and behavioural self-evaluation can improve health outcomes in cardiovascular secondary prevention.

**Supplementary Information:**

The online version contains supplementary material available at https://doi.org/10.1186/s12872-026-06452-x.

## Introduction

Despite substantial advances in pharmacological therapies and revascularisation strategies, cardiovascular disease (CVD) remains a leading cause of morbidity and mortality worldwide [1–3]. In particular, patients with established CVD continue to face a considerable residual risk of recurrent events, even under optimal medical management, including statins, antiplatelet therapy, and guideline-directed interventions [4–6].

While secondary prevention strategies emphasise aggressive risk factor control, including lipid management, blood pressure regulation, and glycaemic control, lifestyle modification remains a cornerstone of long-term risk reduction [7]. However, achieving and sustaining health-related behavioural changes—such as adherence to physical activity, dietary modification, and medication regimens—continues to be a major challenge in clinical practice [8, 9].

Traditional approaches to secondary prevention have largely focused on risk communication, patient education, guideline dissemination, and improving adherence to recommended therapies. Yet, these strategies alone are often insufficient to produce sustained behavioural engagement [8, 9, 12]. Emerging evidence suggests that patients’ beliefs about disease causation—particularly the extent to which they attribute CVD to genetic versus lifestyle factors—may critically influence their perceived control over the disease and, consequently, their motivation to engage in preventive behaviours [10, 11].

These relationships can be understood through behavioural frameworks such as the COM-B model, which conceptualises behaviour as a function of capability, opportunity, and motivation [12]. Within this framework, motivation serves as a central mediator linking cognitive beliefs to behavioural engagement, while psychosocial factors such as self-efficacy further shape the translation of intention into action [13].

Genomic and epidemiological research has demonstrated that both genetic and environmental factors contribute to disease risk [14, 15]. Beliefs about disease causation are often conceptualised along a continuum between genetic determinism and lifestyle attribution [16]. Individuals differ in how they interpret these causal factors, and such differences may have important behavioural implications. Stronger beliefs in genetic causation may be associated with reduced perceived control and lower motivation for behaviour change, whereas stronger beliefs in lifestyle causation may enhance self-efficacy and engagement in health-promoting behaviours [17–19]. However, existing research has focused predominantly on the relationship between illness beliefs and behavioural engagement — that is, whether patients perform health-promoting behaviours. A distinct but underexplored question is whether illness beliefs also shape how patients evaluate the adequacy of their own behaviour — that is, whether patients may be actively engaged in health behaviours and yet perceive their efforts as insufficient. This remains an open empirical question with important implications for understanding persistent gaps in behavioural engagement and for the design of behavioural interventions.

Lifestyle-related factors are recognised as major determinants of cardiovascular risk across populations. A substantial proportion of CVD is attributable to modifiable behavioural and metabolic risk factors [20]. Interventions targeting these factors have been shown to reduce cardiovascular morbidity and mortality, although sustained behaviour change remains inconsistent [21].

These perspectives suggest that patients’ belief systems may represent a critical, yet underexplored, psychological factor in secondary prevention—potentially influencing motivation and engagement in health-related behaviours despite optimal medical therapy.

Therefore, this study aimed to examine (1) cross-national differences in beliefs regarding genetic and lifestyle influences on CVD between patients in the US and Japan, and (2) the associations between these beliefs, exercise confidence, and behavioural self-evaluation. Three hypotheses were proposed: (H1) US patients would show higher genetic attribution and Japanese patients would show higher lifestyle attribution to CVD; (H2) higher genetic attribution would be associated with lower exercise engagement, whereas higher lifestyle attribution would be associated with greater engagement in exercise; and (H3) a significant discrepancy between ideal and actual exercise behaviour would be observed in both countries. Cross-national differences in causal beliefs (H1) were predicted on the basis of documented differences in guideline framing and physician communication practices between the two countries: the 2019 ACC/AHA guidelines explicitly list family history as a key risk-enhancing factor in clinical discussions [29], whereas the JCS 2023 guidelines emphasise lifestyle-related factors as primary intervention targets [30], and US physicians devote significantly more consultation time to patient education than their Japanese counterparts [31].

**Fig. 1:**
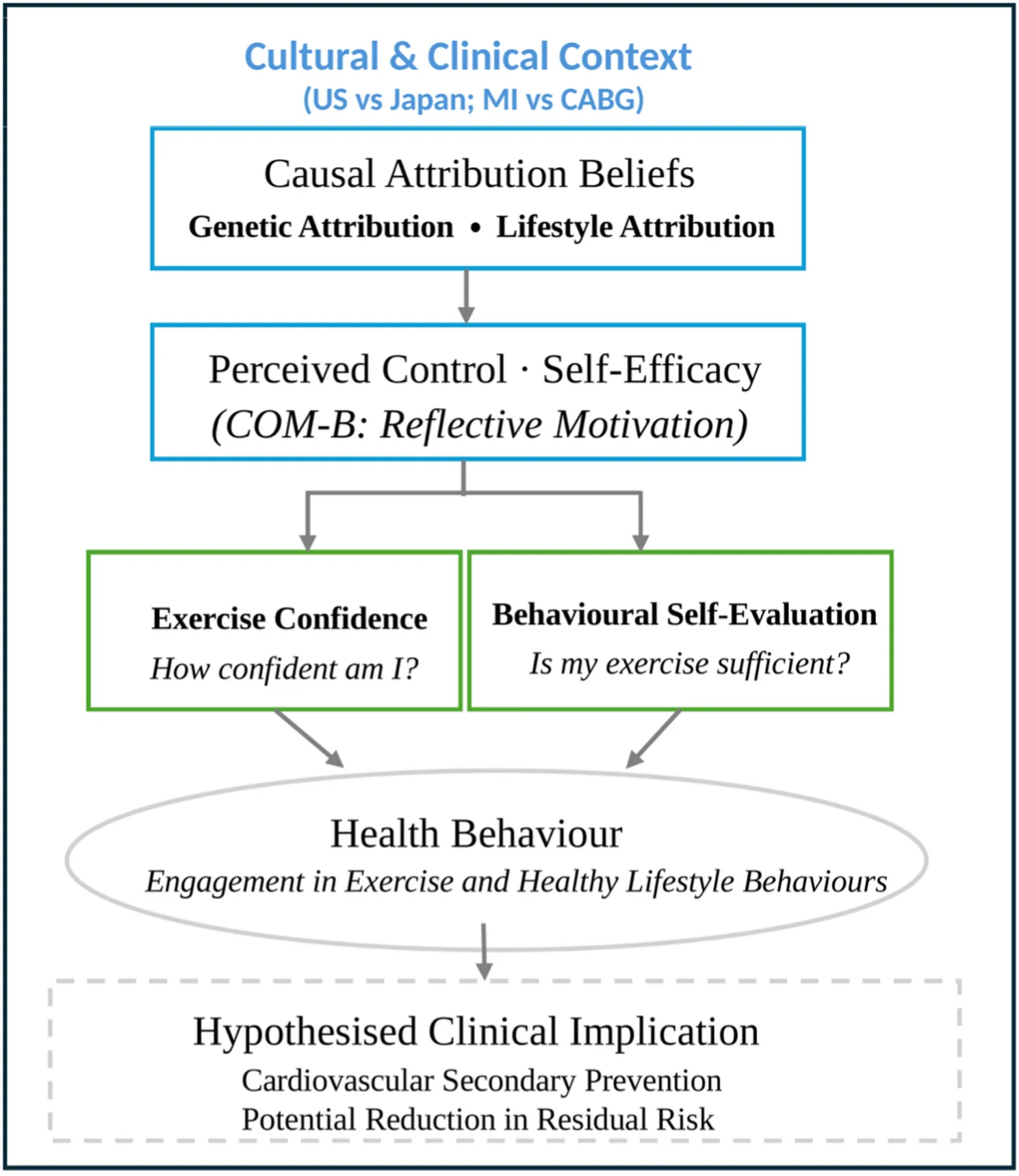
Conceptual framework of the study. Conceptual framework illustrating the hypothesised relationships between causal attribution beliefs, perceived control/self-efficacy (Reflective Motivation), behavioural outcomes, and cardiovascular secondary prevention. The dashed box illustrates a hypothesised clinical implication for cardiovascular secondary prevention

The conceptual framework of this study is presented in Fig. 1.

## Methods

### Study design and participants

This study employed a cross-sectional design using a self-administered questionnaire. Participants were individuals with CVD who had experienced myocardial infarction (MI) or undergone coronary artery bypass grafting (CABG) and were enrolled in phase II cardiac rehabilitation programmes at Scripps Memorial Hospital, La Jolla, California (US) and the National Cerebral and Cardiovascular Center Hospital, Osaka (Japan). Eligibility required participants to be aged 18 years or older, to have participated in the phase II programme on at least four sessions, and to be able to read and write in English (US) or Japanese (Japan).

Participants were recruited between April and September 2015 in the US and between May and June 2015 in Japan. In the US, both paper-based and online questionnaires were administered; in Japan, paper-based questionnaires only were used. Participation was voluntary. A total of 176 participants were enrolled (US: *n* = 56; Japan: *n* = 120). The response rate in Japan was 60% (120 of 200 questionnaires distributed). A response rate could not be calculated for the US cohort because the total number of eligible patients approached was not documented. Recruitment was facilitated by cardiac rehabilitation staff using both paper-based and online questionnaires. Therefore, the reported response rate applies only to the Japanese cohort.

### Measures

The questionnaire was developed by the author specifically for this study, took approximately 30 min to complete, and has not been previously published. An English version is provided as Supplementary Material 1. Item development was informed by the author’s clinical experience facilitating a weekly class for cardiac rehabilitation participants in the US, in which diet and physical activity were routinely discussed. Prior to administration, the questionnaire was reviewed by the programme managers and cardiac rehabilitation nurses at both participating centres and by the author’s doctoral dissertation committee to assess its clinical relevance, clarity, and face validity. No formal psychometric validation (e.g., factor analysis or test–retest reliability) was undertaken.　Beliefs about disease causation were assessed by asking participants to rate, on a scale of 0% to 100%, the extent to which they believed their cardiac condition was caused by genetic factors and by lifestyle factors. For regression analyses, responses were dichotomised into high (≥ 70%) and low (< 70%) attribution groups. Exercise-related outcomes included confidence in achieving ideal exercise goals (five-point ordinal scale: 1 = fairly confident, 5 = not confident at all; lower scores indicate greater confidence) and self-reported exercise sufficiency (Yes/No). The questionnaire included additional items assessing related psychosocial and behavioural factors; however, the present analysis focused on causal beliefs, exercise confidence, and self-reported exercise behaviour, which were most directly aligned with the study aims.

### Statistical analysis

Statistical analyses were performed using IBM SPSS Statistics Version 22 (IBM Corp., Armonk, NY, USA). Descriptive statistics were calculated for demographic characteristics and study variables. Continuous variables are presented as means ± standard deviations (SD), and categorical variables as frequencies and percentages. Before conducting group comparisons of causal attribution scores, normality was assessed using the Shapiro–Wilk test. As all groups showed significant departures from normality (all *p* < 0.01), independent-samples t-tests were complemented by Mann–Whitney U tests as a sensitivity analysis. Both methods yielded consistent conclusions; therefore, t-test results are presented in the main tables.　Participants rated the perceived contribution of genetic and lifestyle factors to their CVD on 0–100% scales. Consistent with the original study protocol, these variables were dichotomised into High (≥ 70%) and Low (< 70%) attribution groups. This threshold was specified a priori in consultation with a statistician before analysis and was intended to represent relatively strong attribution to a single causal factor. Because no established cut-off exists in the illness perception literature, the choice was based on expert judgement rather than previous validation.　Exercise confidence was analysed using a four-factor full factorial ANOVA with country (US/Japan), CVD type (MI/CABG), high versus low genetic attribution, and high versus low lifestyle attribution as fixed factors. The full factorial model included all main effects and all two-, three-, and four-way interaction terms. Type I (sequential) sums of squares are reported, and partial eta-squared (η²) is reported as the effect-size measure. The binary outcome perceived exercise sufficiency (Yes/No) was analysed using binary logistic regression. The model included the four main effects together with all prespecified two-way interaction terms (Country × Disease Type, Country × Genetic Attribution, Country × Lifestyle Attribution, Disease Type × Genetic Attribution, Disease Type × Lifestyle Attribution, and Genetic Attribution × Lifestyle Attribution). Odds ratios (ORs) with 95% confidence intervals (CIs) were reported. Because none of the prespecified two-way interaction terms reached statistical significance, only the main-effects estimates are presented in the main text for ease of interpretation; the full model including all interaction terms is provided in Supplementary Material 2. Missing data were handled using listwise deletion for each analysis; consequently, the number of participants included varied according to the variables required for each statistical model. Cases with missing data on any included variable were excluded from the respective analysis, resulting in an analytic sample of *n* = 150 for the ANOVA and *n* = 141 for the logistic regression. A two-sided *p*-value < 0.05 was considered statistically significant. A sample size of 50 per group provides high statistical power (*β* = 0.96) for detecting strong effects in correlational analyses (*r* = 0.5). The present study exceeded this threshold (US: *n* = 56; Japan: *n* = 120). Samples of similar size have been used in prior cross-sectional studies of cardiac patients [32, 33].

### Ethical approval

This study was approved by Institutional Review Boards in both the US and Japan. Ethical approval was obtained from the National Cerebral and Cardiovascular Center (Approval No. M26-155, Japan) and the Saybrook University Institutional Review Board (Approval No. 15-S-TShirai, United States). The study was conducted in accordance with the Declaration of Helsinki. Written informed consent was obtained from all participants.

## Results

### Baseline characteristics

A total of 176 participants with CVD were included in the analysis (US: *n* = 56; Japan: *n* = 120). Participants were enrolled in phase II cardiac rehabilitation programmes and had a history of MI or CABG. Baseline demographic and clinical characteristics are presented in Table 1.

**Table 1:** Baseline characteristics of study participants

Variable		United States (*n* = 56)	Japan (*n* = 120)
Age, years		66.5 ± 12.0 (45–85)	71.0 ± 9.3 (42–88)
Female, *n* (%)		21 (37.5)	18 (15.0)
Myocardial infarction (MI), *n *(%)		36 (64.3)	66 (55.0)
Coronary artery bypass graft (CABG), *n* (%)		19 (33.9)	54 (45.0)
Race, *n* (%)	Caucasian	42 (75.0)	0 (0.0)
	Latino/Hispanic	5 (8.9)	0 (0.0)
	Asian	4 (7.1)	120 (100)
	Others	2 (3.6)	0 (0.0)
	Native American	1 (1.8)	0 (0.0)
Education, *n* (%)	Graduate School	27 (48.2)	2 (1.7)
	College	19 (33.9)	50 (41.7)
	Technical School	5 (8.9)	15 (12.5)
	High School	4 (7.1)	45 (37.5)
	Junior High School	0 (0.0)	8 (6.7)
Work status, *n* (%)	Retired	32 (57.1)	32 (27.4)
	Working Full-Time	15 (26.8)	24 (20.5)
	Working Part-Time	4 (7.1)	8 (6.8)
	Not Working	4 (7.1)	54 (46.2)
Living with others, *n* (%)		46 (82.1)	112 (92.5)

Percentages are based on available data. Some participants had both MI and CABG and are included in both categories; therefore, disease type percentages may exceed 100%. Missing data were present for some variables (e.g., work status: Japan *n* = 117). Percentages may not sum to 100% due to rounding or missing data. Values for age are presented as mean ± standard deviation (range). SD = standard deviation; MI = myocardial infarction; CABG = coronary artery bypass graft

### Cross-national differences in causal beliefs

Participants were asked to rate, on a scale of 0% to 100%, the extent to which they believed their cardiac condition was caused by genetic factors and lifestyle factors, respectively. Significant cross-national differences were observed in both domains. US participants reported higher genetic attribution than Japanese participants (mean ± SD: 55.33 ± 32.06% vs. 29.36 ± 30.68%; *t*(156) = 4.85, *p* < 0.001; Cohen’s *d* = 0.83), whereas Japanese participants reported higher lifestyle attribution than US participants (63.55 ± 29.62% vs. 41.82 ± 30.28%; *t*(154) = − 4.22, *p* < 0.001; Cohen’s *d* = 0.73). Mann–Whitney U tests yielded results consistent with the independent-samples *t-*tests (Table 2). These findings indicate that US participants attributed their CVD more strongly to genetic factors, while Japanese participants attributed their CVD more strongly to lifestyle factors.

**Table 2:** Perceived genetic and lifestyle attribution to cardiovascular disease by country

Variable	United States	Japan	Cohen’s *d*	Mann–Whitney U	*r*	*p*
Genetic attribution (%)						
Mean ± SD	55.33 ± 32.06	29.36 ± 30.68	0.83	3840.5	0.44	< 0.001
Median (IQR)	50 (35–80)	20 (0–50)				
Lifestyle attribution (%)						
Mean ± SD	41.82 ± 30.28	63.55 ± 29.62	0.73	1571.0	0.40	< 0.001
Median (IQR)	50 (20–55)	70 (50–90)				

All reported statistics — means, standard deviations, t-tests, Cohen’s *d*, medians, IQRs, Mann–Whitney U statistics, and effect size *r* are based on participants with valid attribution data (US *n* = 49; Japan *n* = 109 for genetic attribution and *n* = 107 for lifestyle attribution; values coded as 999 were treated as missing). Cohen’s *d* represents the standardized mean difference based on pooled standard deviations. The *p*-values shown refer to the independent-samples *t*-test; Mann–Whitney U tests yielded consistent results for both variables. SD = standard deviation; IQR = interquartile range; *r* = effect size for the Mann–Whitney U test

### Confidence in achieving exercise goals

Confidence in achieving ideal exercise goals was analysed using a four-factor full factorial ANOVA with country (US/Japan), type of CVD (MI/CABG), perceived genetic attribution (high ≥ 70% vs. low < 70%), and perceived lifestyle attribution (high ≥ 70% vs. low < 70%) as between-subjects fixed factors, including all main effects and interaction terms. A significant main effect of country was observed (F(1,136) = 28.34, *p* < 0.001), with US participants reporting significantly higher confidence in achieving exercise goals than Japanese participants (mean ± SD: 1.42 ± 0.78 vs. 2.29 ± 1.03; lower scores indicate greater confidence). An exploratory interaction was also observed between type of CVD and genetic attribution (F(1,136) = 4.58, *p* = 0.03). Cell sizes were small (MI × High: *n* = 13; CABG × High: *n* = 11); results should be interpreted with caution (Table 3).

**Table 3:** Effects of country, disease type, genetic attribution, and lifestyle attribution on exercise confidence: factorial ANOVA

	*N*	Mean ± SD	*P*
Country			< 0.001
US	52	1.42 ± 0.78	
Japan	98	2.29 ± 1.03	
Type of CVD			NS
MI	81	1.85 ± 0.85	
CABG	61	1.90 ± 1.04	
Genetic attribution			NS
High (≥ 70%)	28	1.64 ± 0.99	
Low (< 70%)	114	1.93 ± 0.92	
Lifestyle attribution			NS
High (≥ 70%)	52	2.02 ± 0.87	
Low (< 70%)	90	1.79 ± 0.97	
Interaction: Type of CVD × Genetic attribution (high ≥ 70% vs. low < 70%)		F(1,136) = 4.58	0.03

Exercise confidence was measured on a five-point ordinal scale (1 = fairly confident, 5 = not confident at all; lower scores indicate greater confidence). Factorial ANOVA was conducted with country (US/Japan), type of CVD (MI vs. CABG), genetic attribution (high ≥ 70% vs. low < 70%), and lifestyle attribution (high ≥ 70% vs. low < 70%) as between-subjects fixed factors in a full factorial model. *SD*  standard deviation, *CVD*  cardiovascular disease, *MI * myocardial infarction, *CABG*  coronary artery bypass graft, *ANOVA*  analysis of variance, *NS*  not significant. The *p*-value reported for Country represents the main effect of country in the full factorial ANOVA

### Discrepancy between ideal and actual exercise behaviour

A significant discrepancy between ideal and actual exercise levels was observed in both countries (US: mean difference = 0.63, *p* < 0.001, Cohen’s *d *= 0.89; Japan: mean difference = 0.33, *p* < 0.001, Cohen’s *d* = 0.39), indicating a consistent gap between participants’ exercise aspirations and their actual behaviour regardless of country.

### Association between causal beliefs and exercise behaviour

Whether participants perceived their current exercise level as sufficient was analysed using binary logistic regression, with country (US/Japan), type of CVD (MI/CABG), perceived genetic attribution (high ≥ 70% vs. low < 70%), and perceived lifestyle attribution (high ≥ 70% vs. low < 70%) as predictor variables, including all main effects and all two-way interaction terms. In the logistic regression analysis, none of the prespecified two-way interaction terms was statistically significant; the main-effects estimates are therefore presented (Table 4). Lifestyle attribution ≥ 70% was the only significant predictor of perceived exercise sufficiency (OR 0.46, 95% CI 0.22–0.97, *p* = 0.042; Table 4). Complete regression output, including the model with interaction terms, is provided in Supplementary Material 2.

No significant associations were observed between causal beliefs and confidence in achieving an ideal diet, nor between causal beliefs and perceived dietary adequacy.

**Table 4:** Logistic regression: predictors of self-reported exercise sufficiency

Part A. Descriptive statistics: proportion reporting sufficient exercise						
**Group**			***N***	**Proportion reporting sufficient exercise**		
Country: US			46	47.8%		
Country: Japan			95	42.1%		
CVD type: MI			83	42.2%		
CVD type: CABG			58	46.6%		
Genetic attribution High (≥ 70%)			25	48.0%		
Genetic attribution Low (< 70%)			116	43.1%		
Lifestyle attribution High (≥ 70%)			57	33.3%		
Lifestyle attribution Low (< 70%)			84	51.2%		

Part B. Logistic regression: main-effects estimates derived from the prespecified full model, *n* = 141						
Variable	B	SE	OR	CI lower	95% CI upper	*p*
Intercept	−0.042	0.320	0.959	0.512	1.797	0.896
Country (US vs. Japan)	−0.040	0.409	0.961	0.431	2.142	0.923
CVD type (CABG vs. MI)	0.261	0.355	1.298	0.647	2.603	0.462
Genetic attribution ≥ 70%	0.053	0.475	1.055	0.415	2.678	0.911
Lifestyle attribution ≥ 70%	−0.778	0.383	0.46	0.22	0.97	0.042

Part A: proportions are based on the logistic regression analysis sample (*n* = 141). Part B: dependent variable = self-reported exercise sufficiency (Yes/No); reference categories: Japan, MI, attribution < 70%. A main-effects logistic regression model is presented (reference categories: Japan, MI, attribution < 70%). All prespecified two-way interaction terms were tested and were non-significant (all *p* > 0.3); full output is provided in Supplementary Material 2. *B* = unstandardised logistic regression coefficient, *SE* standard error, *OR* odds ratio, *CI* confidence interval, *CVD* cardiovascular disease, *MI* myocardial infarction, *CABG* coronary artery bypass graft

## Discussion

This study examined cross-national differences in beliefs about the causes of CVD and their associations with behavioural engagement and perceived self-efficacy among patients participating in cardiac rehabilitation programmes in the US and Japan. H1 and H3 were broadly supported. H2, however, was not supported: no significant direct association was observed between causal attribution and exercise engagement. In the course of these analyses, an additional finding emerged: patients with stronger lifestyle attribution were significantly less likely to report their current exercise level as sufficient. Although this observation was not pre-specified as a hypothesis, it represents an important finding that extends current understanding of how illness beliefs shape health-related cognition and provides a basis for future hypothesis-driven investigation.

CVD accounts for approximately one-fifth of all deaths in the US, where it represents the leading cause of mortality, compared with a lower proportion in Japan, where it is the second leading cause of death [34, 35]. These differences in disease burden are consistent with the divergent guideline frameworks and physician communication practices discussed in the Cultural context section below, and may help explain the cross-national differences in causal beliefs observed in the present study. The present findings further suggest that culturally shaped beliefs may influence not only health-related behaviour, but also how individuals evaluate the adequacy of their own efforts.

### Principal findings

H1 was supported: participants in the US were more likely to attribute their CVD to genetic factors, whereas participants in Japan were more likely to attribute their condition to lifestyle factors. These differences were statistically significant and of moderate effect size, indicating clinical relevance. H2 was not supported: no significant main effects of genetic or lifestyle attribution on exercise confidence or exercise engagement were observed. The only significant effect in the ANOVA was an exploratory interaction between disease type and genetic attribution (F(1,136) = 4.58, *p* = 0.03), suggesting that the relationship between genetic beliefs and perceived control may vary according to clinical experience. H3 was supported: a consistent discrepancy between ideal and actual exercise behaviour was observed across both countries, with a larger gap in the US (Cohen’s *d* = 0.89) than in Japan (Cohen’s *d* = 0.39). An unexpected post-hoc finding was that participants with high lifestyle attribution were significantly less likely to report their current exercise level as sufficient (OR 0.46, 95% CI 0.22–0.97, *p* = 0.042). This may reflect higher personal standards for exercise among those with stronger lifestyle beliefs, suggesting that beliefs influence how patients evaluate the adequacy of their behaviour rather than behavioural engagement itself. Although this finding was not hypothesised a priori, it is consistent with theoretical models of self-regulation and may represent an important extension of existing behavioural frameworks. This distinction is clinically important for goal-setting and behavioural medicine interventions in cardiac rehabilitation: patients who are behaviourally active may nonetheless perceive themselves as falling short, underscoring the need for clinicians to address not only behaviour but also patients’ evaluative standards.

### Interpretation of findings: belief as a mechanism

These findings suggest that the relationship between illness beliefs and health behaviour is more complex than a straightforward belief-engagement pathway. Individuals who perceive their condition as primarily influenced by lifestyle factors may experience a greater sense of personal control and responsibility, which, rather than simply increasing engagement, may simultaneously raise their standards for what constitutes adequate behaviour. This pattern may help explain why high lifestyle attribution was associated with a lower likelihood of perceiving current exercise as adequate (OR 0.46), suggesting that, regardless of actual behaviour, stronger lifestyle beliefs may raise the standard against which individuals evaluate their own behaviour rather than directly increasing behavioural engagement. Within the illness perception literature, the role of causal beliefs in shaping behavioural self-evaluation remains largely unexamined and warrants further investigation. In contrast, theoretical frameworks suggest that individuals who attribute their condition primarily to genetic factors may adopt a more deterministic perspective [18], potentially reducing personal accountability and lowering the standard against which their own behaviour is evaluated, though this was not directly supported in the present study.

Regarding the measurement constructs underlying these findings, it is worth noting what each measure captures at the conceptual level. Causal attribution, assessed as percentage attribution to genetic and lifestyle factors respectively, is conceptually consistent with the causal subscale of the Illness Perception Questionnaire–Revised (IPQ-R) [36], providing theoretical grounding despite the use of a study-specific instrument. Exercise confidence, assessed on a five-point ordinal scale, operationalises the construct of exercise self-efficacy as described by Bandura [13, 19], consistent with established theoretical frameworks of health behaviour. Exercise sufficiency — whether participants perceived their current exercise as adequate — captures a construct that has not been formally operationalised in prior illness perception or self-efficacy research. This measure was not designed to assess behavioural engagement per se, but rather patients’ evaluative judgement of their own behaviour relative to a personal standard. The distinction between these two constructs — engagement and self-evaluation — may be theoretically important and represents a direction for future measurement development. The common-sense model of illness representations proposes that individuals’ beliefs about disease causation shape coping behaviours and health outcomes [22]. Studies on genetic essentialism indicate that stronger beliefs in genetic causation may lead to more deterministic thinking and reduced motivation for behaviour change [18]. Conversely, higher self-efficacy has been consistently associated with greater engagement in health-promoting behaviours [13]. Meta-analytic evidence further supports that illness perceptions are associated with behavioural outcomes, particularly through perceived controllability and personal responsibility [23, 24]. However, the relationship between genetic beliefs and behaviour is not universally negative; some studies suggest that communicating genetic risk can motivate preventive behaviours when appropriately framed [25].

These theoretical pathways were not directly supported by significant main effects in the present study; however, the observed patterns are broadly consistent with this framework. US participants, who more strongly attributed their condition to genetic factors, reported higher exercise confidence than Japanese participants, who more strongly attributed their condition to lifestyle factors. This pattern may reflect the influence of cultural and communicative context on how causal beliefs translate into perceived control, rather than a straightforward individual-level relationship between genetic attribution and reduced motivation. Future longitudinal studies are needed to clarify the directional relationships between causal beliefs, perceived control, and behavioural engagement.

### Integration with the COM-B framework

These relationships can be understood within the COM-B model, in which behaviour is conceptualised as a function of capability, opportunity, and motivation [12]. Within this framework, causal beliefs are theorised to primarily influence the motivational component of behaviour through perceived control and self-efficacy. Stronger lifestyle attribution may enhance reflective motivation by increasing perceived agency, whereas stronger genetic attribution may foster more deterministic interpretations of disease risk. These findings highlight that belief systems may represent a potentially modifiable pathway for improving behaviour change outcomes.

### Interaction between belief and clinical context

One exploratory finding of this study is the interaction between genetic attribution and type of CVD (*F*(1,136) = 4.58, *p* = 0.03). The relationship between genetic beliefs and exercise confidence differed between participants with MI and those who underwent CABG, suggesting that clinical experience shapes perceived control. Among participants with high genetic attribution, those with MI reported relatively higher exercise confidence than CABG patients. Although this interaction reached statistical significance, its interpretation should remain cautious because of the relatively small subgroup sizes. The observed pattern is therefore best regarded as hypothesis-generating pending replication in a larger sample. MI patients recovering from percutaneous coronary intervention (PCI) may experience reduced perceived vulnerability as symptoms resolve [26], whereas CABG patients may be more susceptible to fatalistic thinking when genetic beliefs are strong [27]. Perceived control has been identified as a key determinant of cardiac rehabilitation engagement [28]. These findings suggest that the influence of causal beliefs may vary according to clinical context, whereas lifestyle attribution appears to be more consistently related to behavioural self-evaluation.

### Cultural context

The observed cross-national differences may partly reflect divergent emphases in national clinical guidelines and physician communication practices. The 2019 ACC/AHA guidelines on CVD prevention explicitly list family history as a key risk-enhancing factor in clinician–patient risk discussions [29], whereas the JCS 2023 guidelines on primary prevention of coronary artery disease emphasise lifestyle-related factors such as dietary habits and hypertension as the primary targets for intervention, noting that the lower coronary artery disease burden in Japan compared with Western nations is attributable to differences in lifestyle and risk factor distribution [30]. Furthermore, research comparing physician practice patterns in the two countries has shown that US physicians devote significantly more time to patient education during consultations than Japanese physicians and are more likely to follow practice guidelines routinely [31]. These differences in guideline framing and clinical communication practices are likely to contribute to shaping how patients in each country interpret the causes of their condition. The present findings further suggest that culturally shaped beliefs may influence behavioural self-evaluation—that is, how patients judge the adequacy of their own behaviour. This may represent one mechanism through which cultural differences shape behavioural self-evaluation, even among patients who are already engaging in recommended health behaviours, with potential implications for cardiovascular secondary prevention.

### Implications for clinical practice and public health

The findings carry several specific implications for clinical practice. First, the observed cross-national differences in causal beliefs suggest that clinician–patient communication about disease causation should not be uniform across cultural contexts. Patients in the US may benefit from communication that contextualises genetic risk within a modifiable lifestyle framework, reinforcing perceived control; patients in Japan, who more strongly attribute disease to lifestyle, may benefit from acknowledgement of genetic contributions to support a more balanced causal attribution. Second, the interaction between disease type and genetic attribution suggests that communication strategies should be tailored to patients’ clinical trajectory: among patients with high genetic attribution, those recovering from CABG may require particular attention to perceived control and motivation, whereas MI patients may be at risk of overconfidence that reduces engagement. Third, and perhaps most importantly, the post-hoc finding that lifestyle attribution was associated with lower perceived exercise sufficiency suggests that clinicians should assess not only whether patients are engaging in health behaviours, but also how patients evaluate the adequacy of their own efforts. Patients who are behaviourally active but chronically perceive themselves as falling short may be at risk for maladaptive outcomes and may benefit from interventions that recalibrate personal standards rather than simply increase behavioural engagement.

## Limitations

Several limitations should be considered. First, the cross-sectional design was appropriate for the aim of characterising the prevalence and pattern of illness beliefs and their concurrent associations with behavioural outcomes, but precludes causal inference; longitudinal designs are needed to examine whether changes in beliefs precede changes in behaviour. The logistic regression model included multiple predictors and interaction terms relative to the available sample size, and results should therefore be interpreted with caution. Although the interaction terms were not statistically significant and are reported only in the Supplementary Material, the limited sample size may have reduced power to detect interaction effects. The wide confidence interval observed for the primary logistic regression finding (OR 0.46, 95% CI 0.22–0.97) further reflects the statistical uncertainty associated with the modest sample size.

Second, participants were recruited from cardiac rehabilitation programmes at single urban centres in each country, and the observed differences may therefore reflect site-specific rather than national variation. In addition, patients enrolled in Phase II cardiac rehabilitation may represent a relatively motivated subgroup of the broader CVD population, as programme participation requires referral, access, and sustained attendance. The present findings may therefore not generalise to patients who decline or discontinue rehabilitation.

Third, the measures used in this study were developed specifically for this cross-national comparison and were not formally validated. Causal attribution was assessed using a 0–100% rating scale for genetic and lifestyle factors, which is conceptually consistent with the causal subscale of the IPQ-R [36], though psychometric properties such as test-retest reliability were not established. For regression analyses, responses were dichotomised using a threshold of 70%, determined a priori based on statistical consultation, with the rationale that attribution of 70% or greater to a single factor would indicate a predominantly single-factor causal belief. As no established cutoff exists in the illness perception literature, the sensitivity of findings to alternative thresholds was not examined. Exercise confidence was assessed using a single five-point ordinal item operationalising exercise self-efficacy [13], rather than a validated multi-item scale such as the Cardiac Exercise Self-Efficacy Instrument. Exercise sufficiency was assessed using a single binary item capturing patients’ self-evaluation of their own behaviour; no established measure of this construct currently exists, and the present operationalisation should be considered a preliminary step toward formal scale development. However, the absence of objective measures of physical activity limits the ability to distinguish between actual behavioural insufficiency and differences in perceived standards. Future research incorporating objective assessments of physical activity may help clarify whether lower perceived adequacy reflects behavioural differences or more stringent self-evaluation. Finally, the data were collected in 2015; however, beliefs about disease causation are relatively stable constructs.

## Conclusion

This study demonstrates that beliefs about the causes of CVD differ significantly between the US and Japan and are associated with exercise confidence. These findings suggest that patients’ belief systems may represent an important psychological pathway through which individuals perceive and evaluate their own health behaviours, with potential implications for gaps in behavioural engagement despite advances in medical therapy. Clinician–patient communication may play a role in shaping these beliefs. Balanced communication that acknowledges genetic risk while reinforcing modifiable lifestyle factors may enhance perceived control and support engagement in health-promoting behaviours. These findings provide hypothesis-generating evidence for the development of translational interventions targeting belief systems in cardiovascular secondary prevention. Behavioural adequacy may represent an independent target for behavioural intervention, distinct from behavioural engagement itself — a distinction that has not been formally examined in the illness perception literature and warrants dedicated investigation. Future longitudinal and intervention studies are warranted to determine whether changes in causal beliefs and behavioural self-evaluation are associated with improved health outcomes in cardiovascular secondary prevention.

## Supplementary Information

Supplementary Material 1.

Supplementary Material 2.

## Data Availability

The datasets generated and analysed during the current study are available from the corresponding author upon reasonable request.
