# Supplementary material for: Causal beliefs and behavioural self-evaluation in cardiac rehabilitation: a cross-national study in the United States and Japan
Source: BMC Cardiovasc Disord. 2026 Aug 10;26:1034. doi: 10.1186/s12872-026-06452-x (PMC13737510; doi:10.1186/s12872-026-06452-x)
Supplement: Supplementary file 1 [file 12872_2026_6452_MOESM1_ESM.docx]

**Supplementary Material 1. Questionnaire (English version)**

Please answer the following questions.


| **1 Gender:** M or F | **2 Age:**  __________ Years old |  |
| --- | --- | --- |
| **4 Height:** __________ Inch | **5 Weight:** __________ Pound | **6 BMI:** (if you know) |
| **7 Race:** (Circle a number.)  1. African American 2. Asian 3. Caucasian 4. Latino/Hispanic 5. Native American  6. Pacific Islander 7. Other ( ) 8. No answer | | |
| **8 Last education:** (Circle a number.)  1. Elementary school 2. Junior high school 3. High school  4. Technical school/Community college 5. College 6. Graduate school | | |
| **9 Working situation:**  1. Full-time working 2. Part-time working 3. Retired 4. Not working | | |
| **10 Do you currently live with anyone?**  Yes No | **11 When did you last come to Cardiac Rehab?**  Month __________ Date __________ Year __________ | |

**B.**

| **1 What made you come to Cardiac Rehab?** (Circle the number(s) that apply.)  1 Congenital heart disease 9 Atrial fibrillation  2 Coronary heart disease 10 Ischemic heart disease  3 Dyslipidemia 11 Angina pectoris  4 Hypertension 12 Cardiomyopathy  5 Atherosclerosis 13 Dissection of aorta  6 Valvular disease of the heart 14 Stroke  7 Rheumatic heart disease 15 Post-surgery recovery  8 Arrhythmia 16 Others (specify the reason: ) | |
| --- | --- |
| **2 Tell us about your condition in more detail.** (Write in this space.) | |
| **3 Do you also have these chronic conditions and receive treatment for them?** (Circle the number(s) that apply.) | 1 Type 2 diabetes mellitus  2 High cholesterol  3 Chronic obstructive pulmonary disease (COPD)  4 Arthritis  5 Depression, Anxiety  6 Osteoporosis  7 Cancer  8 Others (Please specify: )  0 I don't have any other chronic conditions |

**C.**

| **1 Are you currently participating in gym exercise or classes of any kind at a Cardiac Rehabilitation facility?**  If **Yes**, select options you are participating in, and if **No**, please select reasons why you don't participate from the below options. | Yes No |
| --- | --- |
| **2 If "Yes," indicate the activities in which you are participating: (Circle all relevant numbers.)**  1 Gym exercise  2 Nutrition class  3 Education class  4 Support group  5 Yoga, Tai Chi, Meditation, Music therapy  6 Others (specify: ) | |
| **3 If "No," indicate the reason(s) why you don't participate: (Circle all relevant numbers.)**  1 Physical condition does not allow for participation  2 Financial condition does not allow for participation  3 Schedule does not allow for participation  4 Found other self-care options  5 Other reasons (specify: ) | |
| **4 When did you start Cardiac Rehabilitation?** Month __________ Day __________ Year __________ | |

**D. Please tell us about your experience with exercise.**

| **1 In the last four weeks how many days did you exercise for your health on average per week including cardiac rehab and other exercises?**  __________ Day(s) per week | **3 What do you consider to be your ideal amount of exercise for your health?**  __________ Day(s) per week __________ Hour(s) per week |
| --- | --- |
| **2 How long in total have you done exercise for your health on average per week over the last four weeks?**  __________ Hour(s) per week | **4 How confident are you about achieving this exercise goal? (choose one from the below numbers.)**  1 I am pretty confident I will achieve it  2 I am somewhat confident I will achieve it  3 I am neither confident nor unconfident  4 I am somewhat sure I will not achieve it  5 I am sure I will not achieve it |
| **5 Do you think you get enough exercise?** Yes No  (If 'Yes,' then go to D2, if 'No' go to the below question.) | |

**6 What are your main obstacles in not gaining enough exercise? (Check all the relevant reasons from the below list.)**

1	Your own physical condition (pain, fatigue etc.)

2	Your own motivational issue (laziness, don't feel like doing exercise etc.)

3	Your own motivational issues at the venue (don't want to have exercise with other people, don't like exercise programs or the venue etc.)

4	Financial issues (cannot afford gym exercise or cardiac rehab etc.)

5	Time pressures or your schedule do not allow you to exercise (work, family commitment etc.)

6	Weather, temperature extremes

7	Distance, commuting issues to the venue

8	Other (Please specify: )

**7 Please list the main 3 reasons why you don't get enough exercise in order or importance using the numbers (1-8) from the above list.**

1. __________________	2. __________________	3. __________________

**8 Please tell us what objective you care most about in doing your daily exercise? Do you have any special exercise for improving or protecting your health?**

**D2**

| **1 Do you think you have enough knowledge about exercise in order to achieve good health through exercise?**  Yes No  (if "No" go to the right question) | **2 What kind of knowledge about exercise do you feel you lack in order to achieve good health?** |
| --- | --- |
| **3 From where do you obtain knowledge and information about exercise? Choose all relevant items from the below list.**  1 Healthcare professionals including the cardiac rehab facility  2 Community (neighbors, group of people for hobbies, community center in the area etc.)  3 TV or radio  4 Books, magazines  5 Internet  6 Friends or family members  7 Others (Please specify: ) | |
| **4 Please list the 3 main resources you use for exercise information in order of importance using the above numbers (1-7).**  1. __________________ 2. __________________ 3. __________________ | |
| **5 If you have changed your exercise routine, what was the single source of information that was most influential in making this change (also, from where did you obtain this information): (For example, I started weight-lifting / influenced by my family member)** | |

**E.**

| **1 Do you want to lose weight?**  Yes No  (If No, go to F)  (If, Yes go to 2 and 3)  2 How much weight do you want to lose?  __________ Pound | **3 How confident are you that you will achieve your weight loss goal? (Circle from one of the below numbers.)**  1 I am pretty confident I will achieve it  2 I am somewhat confident I will achieve it  3 I am neither confident nor unconfident  4 I am somewhat sure I will not achieve it  5 I am sure I will not achieve it |
| --- | --- |

**F.**

**1 To be healthy, what do you do with your diet? Choose all items from the below list which you did in the last four weeks.**

1	I tried to eat more vegetables.

2	I tried to eat more fruit.

3	I tried to eat more meat.

4	I tried to eat more fish.

5	I tried to eat more organic food.

6	I tried to eat more carbohydrates.

7	I tried to eat more protein.

8	I tried to eat more fat.

9	I tried to eat more fiber.

10 I tried to eat more dairy products.

11 I tried to drink more water.

12 I tried to eat a more balanced food among the different nutrition groups.

13 I tried to eat more sodium.

14 I tried to eat more sugar.

15 I tried to eat more processed food.

16 I tried to eat more home-cooked food.

17 I tried to check food nutrition labels before buying food.

18 I tried not to eat too much.

19 I tried to avoid eating vegetables.

20 I tried to avoid eating fruit.

21 I tried to avoid eating meat.

22 I tried to avoid eating fish.

23 I tried to avoid eating organic food.

24 I tried to avoid eating carbohydrates.

25 I tried to avoid eating protein.

26 I tried to avoid eating fat.

27 I tried to avoid eating fiber.

28 I tried to avoid eating dairy products.

29 I tried to avoid drinking too much water.

30 I tried to avoid eating sodium.

31 I tried to avoid eating sugar.

32 I tried to avoid eating processed food.

33 I tried not to skip meals.

34 I tried to avoid desserts and snacks.

35 I tried to avoid sauces and dressings.

36 I tried to avoid eating ready-to-eat meals.

37 I tried to avoid eating fast food.

38 I tried to avoid restaurant food.

39 I tried to avoid eating at mid-night.

40 I tried to avoid drinking alcohol.

**2 Do you take supplement? If Yes, what kind of supplement do you take?**

Yes	No	(supplements)

| **3 What do you consider to be your ideal diet for good health?** | **4 How much are you confident of achieving your ideal diet? (Circle from one of the below numbers.)**  1 I am pretty confident I will achieve it  2 I am somewhat confident I will achieve it  3 I am neither confident nor unconfident  4 I am somewhat sure I will not achieve it  5 I am sure I will not achieve it |
| --- | --- |
| **5 Do you think your diet now is the ideal one for you?** Yes No  (If 'Yes,' then go to F2, if 'No' go to the below question.) | |
| **6 What are your main obstacles in achieving your ideal diet? Choose all relevant reasons from the below list.**  1 Your own physical condition (less appetite, fatigue etc.)  2 Your own motivational issue (laziness, don't like certain food etc.)  3 Financial issues (healthy food is expensive etc.)  4 Time pressures or your schedule do not allow for healthy meals (work, family commitment etc.)  5 Your family member(s) cook for you (no choice over menu etc.)  6 Distance to the organic food store, no access to healthy food stores  7 Other (Please specify: ) | |
| **7 Please list the top 3 reasons or obstacles that prevent you from achieving your ideal diet in order of importance using the above numbers (1-7).**  1. __________________ 2. __________________ 3. __________________ | |

**F2**

| **1 Please tell us what is most important to you in your daily diet? Do you have any special diet for being healthy?** | | |
| --- | --- | --- |
| **2 Do you think you have enough knowledge about your diet to be healthy?**  Yes No  (if "No" go to 3 in the right) | | **3 What kind of knowledge about diet do you feel lack in order to be healthy?** |
| **4 From where do you obtain knowledge and information about healthy diets? Choose all relevant items from the below list.**  1 Healthcare professionals including your cardiac rehab facility  2 Community (neighbors, group of people for hobbies, community center in the area etc.)  3 TV or radio  4 Books, magazines  5 Internet  6 From friends or family members  7 Others (Please specify: ) | | |
| **5 Please list the top 3 resources you use for a healthy diet in order of importance using the above numbers (1-7).**  1. __________________ 2. __________________ 3. __________________  **6 If you have changed your diet, what was the single source of information that was most influential in making this change (also, from where did you obtain this information?): (I changed to Atkins Diet / influenced by a TV program)** | | |
| **F3.** | | |
| **1 Do you use any prescription medicines?**  Yes No (If 'No' go to G) | **2 How strictly do you follow your regimen of prescription medications? (choose one number)**  1 I follow it pretty strictly. (if '1' go to G)  2 I follow it somewhat strictly.  3 I am somewhat lax about following my prescription regimen.  4 I am lax about following my prescription medication. | |
| **3 What are your main reasons for not adhering strictly to your prescription regimen? (Check all the relevant reasons from the below list.)**  1 Your own physical condition (cannot tolerate the medication(s), feel bad when you take the medication(s) etc.)  2 Your own motivation (don't feel that medication helps, have your own other supplements or treatment ideas, concerned about side effects, don't like taking 'unnatural chemicals,' etc.)  3 Your unintentional behavior (forgot to take medication, took wrong dosage mistakenly etc.)  4 Location issue (pharmacy is far away or inconvenient)  5 Financial issues (cannot afford medications)  6 Time pressure or your schedule does not allow you to go to pharmacy  7 Other (Please specify: ) | | |
| **4 Please list the main 3 reasons why you don't adhere strictly to your regimen of prescription medications in order or importance using the numbers 1-7 from the above list.**  1. __________________ 2. __________________ 3. __________________ | | |
| **5 How confident are you that your prescription medications will help your condition? (choose one number)**  1 I am pretty confident my prescription medication will help my condition  2 I am somewhat confident my prescription medication will help my condition  3 I am neither confident nor unconfident my prescription medication will help my condition  4 I am somewhat sure my prescription medication will not help my condition  5 I am sure my prescription medication will not help my condition | | |

**G.**

**1 Have you ever thought that your cardiac condition may be partly the result of your genes? Research tells us that even if we are genetically inclined toward a specific condition, we can maintain/change how our genes are expressed by changing our lifestyle such as stopping tobacco use and reducing alcohol.**

**To what extent do you think your cardiac condition is caused by your genes? And how much is caused by your lifestyle?**

Genetic: ______________________________ % (0% - no influence – 100% caused by genes)

Lifestyle: ______________________________ % (0% - no influence – 100% caused by lifestyle)

**2 What kind of lifestyle do you think has negatively affected your cardiac condition? Choose all relevant items from the below list.**

1	Smoking tobacco

2	Drinking alcohol

3	Inactivity

4	Obesity/Being overweight

5	Food intake

6	Stress

7	Lack of sleep

8	Inconstant life rhythm

9	Personality

10 Environmental influences or toxins

11 Other (Please specify: )

**3 Please list the top 3 lifestyle factors that you believe have affected your medical condition in order of importance using the above numbers (1-11).**

1. __________________	2. __________________	3. __________________

**H.**

**1 After you found you had a cardiac condition, how often did you think about the below issues? (Circle the number from 1. I never thought 2. I rarely thought 3. I sometimes thought 4. I often thought.)**

a. Uncertainty	1	2	3	4

b. Prognosis	1	2	3	4

c. Resuscitation	1	2	3	4

d. Spirituality	1	2	3	4

e. End-of-life preparation	1	2	3	4

**2 After you found you had a cardiac condition, what did you do? Choose all relevant items from the below list.**

1	I learned about my condition.

2	I learned how to keep my condition stable and the potential treatment for my condition.

3	I helped my loved ones understand my condition.

4	I prepared for the possibility of sudden hospitalization or a potential emergency.

5	I looked into the medical costs of my condition and sought ways to reduce medical costs.

6	I sought information on the average life span of people with my condition.

7	I discussed with people I trust my wishes for terminal care or end-of-life treatment.

8	I discussed with people I trust potential conflicts that may occur among my family members/loved ones after my passing.

9	I tried to make my loved ones better able to cope with the uncertainty of my condition.

10 I tried to achieve better communication with my loved ones.

11 I tried to spend more quality time with my loved ones.

12 I tried to find people who can listen to my fears and emotions about my condition.

13 I tried to listen to my loved ones' fears and emotions over my condition.

14 I tried to have meditative/spiritual time in order to seek hope or purpose in life.

15 Other (Please specify: )

16 Other (Please specify: )

**3 Which issue do you think in need to think about and to discuss? With whom you would like to discuss? Please circle from a. to e. in need, and choose relevant number(s).**

a. Uncertainty	1 Doctor	2 Nurse	3 Healthcare professionals	4 Patient group

5 Religious professional	6 Family/Relatives	7 Close friends

8 Others (Specify: )

b. Prognosis	1 Doctor	2 Nurse	3 Healthcare professionals	4 Patient group

5 Religious professional	6 Family/Relatives	7 Close friends

8 Others (Specify: )

c. Resuscitation	1 Doctor	2 Nurse	3 Healthcare professionals	4 Patient group

5 Religious professional	6 Family/Relatives	7 Close friends

8 Others (Specify: )

d. Spirituality	1 Doctor	2 Nurse	3 Healthcare professionals	4 Patient group

5 Religious professional	6 Family/Relatives	7 Close friends

8 Others (Specify: )

e. End-of-life preparation	1 Doctor	2 Nurse	3 Healthcare professionals	4 Patient group

5 Religious professional	6 Family/Relatives	7 Close friends

8 Others (Specify: )

**4 If there are some services in cardiac rehabilitation, which services would you like to receive? Please circle from a to e, and tell us what kind of services would be helpful to support your total well-being in the below blank space.**

a. Uncertainty	b. Prognosis	c. Resuscitation	d. Spirituality	e. End-of-life preparation

Thank you very much.
